# From bare life and necropolitics to a feminist care ethic: ageism in the COVID-19 pandemic and future directions

**DOI:** 10.3389/fsoc.2024.1372926

**Published:** 2024-03-07

**Authors:** Bethany Simmonds

**Affiliations:** Department of Geography and Earth Sciences, Aberystwyth University, Aberystwyth, United Kingdom

**Keywords:** ageism and age-based discrimination, necropolitics, feminist care ethics, bare life, COVID-19, risk, health and social care

## Abstract

This perspective paper begins with discussing how COVID-19 magnified the pre-pandemic ‘bare life’ conditions which exposed older people’s lives to risks and indignities in the health and social care system. Then, by using the concept of Necropolitics, the life and death decisions, based on age as a proxy measure for population health during the pandemic, are discussed. This discussion includes examples of ‘exceptional’ practices that were implemented in the UK during the first wave, including ‘Do Not Resuscitate’ orders, unsafe hospital discharges, not transferring to hospitals, and denying access to treatment for older people. It then goes on to renew the call for a feminist care ethic to be central to the ways in which our future health and social care systems are configured. Arguing for the need to politically reframe ageing, health and social care provision towards a radical alternative system that rethinks care relations and addresses inequality.

## Introduction

When COVID-19 hit in March 2020, the NHS in the UK had gone through the most challenging set of circumstances since its inception in 1948; it occurred at a time when health and social care institutions were underfunded, understaffed, fragmented, and poorly coordinated with each other (Simmonds, 2021). Thus, the ageist practices described in this paper are not unique to the COVID-19 pandemic but did magnify the ‘bare life’ conditions which exposed older people’s lives to risks and indignities in the health and social care system (Waring and Bishop, 2020). The result was devastating for older people in the UK, particularly the tragedy of numerous deaths in care and residential settings.

What did change during the COVID-19 pandemic, however, was the necropolitical decision-making became explicit and exposed in the ‘care’ conditions that some older people found themselves in during the COVID-19 pandemic in the UK, particularly those in residential care settings. For instance, it illuminated a spatialised control of populations, where the state implemented modes of exception in relation to who can live and who can be left to die (Mbembe, 2003). In this paper, I discuss examples of some of the ‘exceptional’ practices which were implemented during the UK’s first COVID-19 pandemic wave, which determined based on age, who could live and who could be left to die. These included inappropriately applied ‘Do Not Resuscitate’ orders, unsafe hospital discharges, not transferring to hospitals, and denying access to treatment.

In this perspective paper, I am advocating for Tronto’s (1993) position that instead of employing universalistic impartial ethical frameworks like utilitarianism to guide decisions about care in a detached distanced manner using standardised protocols, a feminist care ethic needs to be central to the ways in which our future health and social care systems are configured. However, I start by providing the context into which the treatment of older people in the COVID-19 pandemic can be situated.

### 1.1 Bare life: older people in pre-pandemic health and social care systems

Since the 1980s, health and social care systems in the UK, as well as other countries in the Global North, have been neoliberalised and impacted by globalisation inasmuch as welfare state systems have to a greater or lesser degree been undermined by the deregulation of social and working protections, and previously publicly run services have been outsourced to large multinational conglomerates (Simmonds, 2021). The resultant reduction in the quality-of-care services in the UK was significantly affected by ‘caretelisation’ (Scourfield, 2011). Cartelization is where large companies buy up and merge small care organisations (Scourfield, 2011). At a time when more funding should have been provided to address the growing numbers of older people needing care, austerity measures were hitting local authorities with a 21% reduction in funding per person between 2009–10 and 2015–16 meaning social care budgets were cut and eligibility criteria tightened (Harris et al., 2019). During the austerity years, 600,000 fewer older people received social care per year (Darzi, 2018), leaving them at risk of getting stuck in the hospital or facing unsafe discharges and likely readmissions.

Furthermore, Waring and Bishop (2020) highlight how, before the pandemic, older people’s rights in large bureaucratic organisations like hospitals, particularly when being transferred into the community, were being eroded. They found that the social organisation of discharge inadvertently exposed older people’s lives to risks and indignities, which were normalised. However, this was less to do with state power and more to do with the product of complex unworkable systems; nevertheless, they argue that these systems reduced older people’s lives to ‘bare life’ (Agamben, 2005). ‘Bare life’ is the product of the state implementing legal exceptions to the treatment of groups that are not recognised as citizens and therefore can be legitimately killed (Agamben, 2005). ‘Social-cultural organisations’ determine the thresholds for what is considered a life of value and what is not, and these modes of exception have become normalised in institutions, particularly where there is spatialised control of disempowered and disenfranchised populations (Waring and Bishop, 2020). Therefore, even before the pandemic, the lives of older people in the health and social care system in the UK were devalued and put at risk, what changed during the COVID-19 pandemic, is this necropolitical decision-making became explicit and exposed.

## 2 Biopower and necropolitics

Biopolitical power, as theorised by Foucault (1978), describes a shift in the way the state protects the sovereign’s life, from the use of the gallows to protecting the ‘social body’ or general population via disciplines like epidemiology: ‘It is no longer a matter of bringing death into play in the field of sovereignty, but of distributing the living in the domain of value and utility’ (Foucault, 1978: 144). Thus, biopower encapsulates the transfer of power from the sovereign’s right to kill, to managing populations to ensure the survival of the perceived stronger group; therefore, following the industrial revolution, the focus of state power was on control of populations’ health (1978).

Necropolitics (Mbembe, 2003) extends Foucault’s conceptualisation of biopower to argue that the state also implements modes of exception in relation to who can live and who can be left to die. This control over decisions of life and death is enabled via categorisations that mark out those who matter and those who do not, and the differential spatialised control of these segregated populations. Mbembe (2003) argues that biopower is insufficient to capture the techno-spatialised capability of power of late modern state to exert death over the living. Mbembe writes as a Cameroonian scholar living in South Africa, about the impact of colonialism. Thus, in using his theorisation of necropolitics, it is acknowledged that, while both racism and ageism kill people, they are not the same thing. For instance, not all of his theorisation can be applied to the experiences of older people during the pandemic. Although arguably the spatialised control of populations—enabling conditions inferring on people the status of the ‘living dead’ (Mbembe, 2003)—is relevant to the ‘care’ conditions that some older people found themselves in during the COVID-19 pandemic in the UK, particularly those in residential care settings.

Robertson and Travaglia (2020) and Travaglia and Robertson (2021) extended the work of Mbembe (2003) and Waring and Bishop (2020), examining the necropolitical assumptions made in decisions of who got treatment and who did not during the pandemic. They called into question social-cultural assumptions about the value of different groups of lives in times of crisis (Robertson and Travaglia, 2020). Although decisions over treatment based on, what is considered a life of value and what is not, occurred prior to crises like COVID-19, and during the pandemic, utilitarian medical philosophical decision-making was magnified. This is where ‘need’ is assessed based on the overall benefit to society and the extent to which existing chronic health conditions will impede clinical benefit (Robertson and Travaglia, 2020). However, during the pandemic, life and death decisions were based on age as a proxy measure for population health, without considering the social implications, human rights, and dignity of groups at the receiving end of intersecting structures of inequality (Colombo, 2021; Travaglia and Robertson, 2021). What follows are examples of how a state can implement modes of exception, determining who can live and who can be left to die.

### 2.1 Examples of COVID-19 ‘exceptional’ practices

The necropolitical practices that were originally described by Mbembe (2003) are evident in some of the decision-making of British state, particularly during the UK’s first COVID-19 pandemic wave. One of the more publicly discussed discriminatory practices involved discharging older patients into care and residential homes without testing for COVID-19. During a legal challenge in the High Court, lawyers for the Department of Health and Social Care stated that they implemented this policy because they were unaware, at the beginning of the pandemic, of asymptomatic transmission and infections and were not made aware of the dangerous repercussions of discharging older patients into care homes with unknown infection statuses (Booth, 2022). However, asymptomatic transmission and widespread deaths in care homes were widely reported in several other countries, including Spain and Italy, in March 2020 (Amnesty International UK (AIUK), 2020; Horton, 2020) and were discussed by the chief scientific advisor of the government on the radio in mid-March 2020. Although infection procedures were put in place before the second wave in September 2020, the spread within care homes due to these unsafe discharge practices had already taken its toll; by the end of June 2020, 31 per cent of all registered deaths in the UK were in care homes (Bell et al., 2020). In 2022, the High Court ruled the policy to be unlawful (BGS, 2022; Booth, 2022).

Another practice, documented by both Amnesty International UK (AIUK) (2020, 2021) and Calvert and Arbuthnott (2021), involved older people, living in both the community and residential settings, not being transferred to a hospital despite it being clinically necessary. These decisions were based on their ‘older’ age status alone. For example, in the first wave, some ambulance services were advised not to admit any ‘elderly’ [*sic*] patients to the hospital; indeed, some crews saw only a handful of older patients during the peak when, pre-2020, they would be frequently attending patients in this demographic group. Indeed, even when an older patient was admitted to a hospital ward, Calvert and Arbuthnott (2021) report, a ‘score of three domains’ triaging tool was used to ration access to intensive care treatment, including ventilators. Nine points were originally given for being over 80 years old, which was enough to pass the threshold for being refused treatment; however, this was readjusted to allocate more points for existing health conditions and fewer points for being over 80. Nevertheless, there were reports of this revised tool being rigorously applied even when beds and ventilators were available. People over 80 were confined to what one family member called ‘death wards’:

Vivien says that inside there were eight elderly [*sic*] men infected with the virus whom she describes as the ‘living dead’… lying ‘half naked in nappies’ on their beds in stifling heat looking ‘drugged and dazed’. The scene was heart-breaking: ‘To see people just dying, all around you’ (Calvert and Arbuthnott, 2021: 245).

At the time, only 2.5 per cent of those over eighties were provided with intensive care treatment, while 50 per cent of those dying were over 80; however, for those that did receive intensive care treatment, the chance of survival was 40% (Calvert and Arbuthnott, 2021) and those without any chronic health conditions were predicted may have lived for another 7 years (on average) if they had not contracted the virus (Hanlon et al., 2021).

Legal orders put in place to signal someone does not wish to be resuscitated should be discussed with the individual and the family, then agreed upon as part of an advance care plan designed to empower individuals, ensuring their wishes are met at the end of their lives. However, during the COVID-19 pandemic, end-of-life care policies were inappropriately applied to groups of residents based on age and/or because they live in a residential care home (Amnesty International UK (AIUK), 2020, 2021; Care Quality Commission (CQC), 2020, 2021; Stevenson, 2020; Wearmouth, 2020; Calvert and Arbuthnott, 2021). Amnesty International UK (AIUK) (2020, 2021) and Calvert and Arbuthnott (2021) both reported that local councils had asked GP surgeries to search and apply blanket ‘Do Not Resuscitate’ orders for all residents in residential and care home settings. When this was leaked to the media, there was an outcry, and these directives were withdrawn. Although rationing of healthcare based on intersections of age and disability is not unique to the pandemic, it intensified, and in many ways normalised its practice, no longer being seen as ‘exceptional’. In fact, some practices, like inappropriately applied ‘Do Not Resuscitate’ orders, have been left in place (Amnesty International UK (AIUK), 2021), due to a lack of training and the presence of appropriately qualified staff (Care Quality Commission (CQC), 2021). ‘Do Not Resuscitate’ orders have also in some cases been misinterpreted to mean that residents should be denied any medical care, including being taken to hospital (Care Quality Commission (CQC), 2021).

The misuse and misapplication of these end-of-life practices during the pandemic, which are designed to promote, rather than remove agency, have highlighted the ways in which they can be applied to discriminate based on age. They have reduced and rationed health resources, when there would be significant clinical benefit, and when, on average, someone over 80 with no co-morbidities, without contracting COVID-19, may have lived for a further 7 years on average (Hanlon et al., 2021). Nevertheless, as Travaglia and Robertson (2021) state, the utilitarian medical philosophical conceptualisation of ‘need’ is not just based on the individual assessment but on the overall benefit to society, and age has been used as a blunt proxy measure for health. The Equality Act (HM Government, 2010) legislates for age as being a protected characteristic, like gender, disability, and ethnicity, which cannot be used to discriminate. However, when it comes to healthcare provision, age can be used to justify not providing healthcare services, if there is a good rationale for doing so (HM Government, 2010). This presents healthcare providers in the UK with a legal loophole if challenged in the courts. Furthermore, the discriminatory practices discussed in this paper can be seen as examples of how older people in healthcare spaces and times can be seen to inhabit ‘death worlds’, where spatialised control of populations is at the whim of states deciding over their life and death (Mbembe, 2003).

## 3 Discussion: transforming the health and social care system using a feminist care ethic

The concept of bare life (Agamben, 2005) has been used in this paper to highlight the impact that neoliberalising health and social care, then austerity measures, had on normalising institutional decision-making, which risked the lives of older people when discharging them from hospital. This discussion was followed by an examination of necropolitical decision-making (Mbembe, 2003), exposing how modes of exception have been applied to the lives of older people (amplified in the first wave of the COVID-19 pandemic in the UK). Practices that contravene older people’s human rights, such as withdrawing lifesaving treatment via techniques such as triage tools or legal orders originally designed to empower people at the end of their lives, can be seen as examples of the state making decisions about who can live and who can be left to die. Those at the receiving end of these practices were spatially controlled in residential care homes or hospital wards, and policies were employed to ration healthcare underpinned by utilitarian medical philosophy, which argues that need should be assessed on a societal, rather than an individual basis (Robertson and Travaglia, 2020).

In this perspective paper, I am advocating for Tronto’s (1993) position that, instead of employing universalistic impartial ethical frameworks like utilitarianism to guide decisions about care in a detached manner using standardised protocols, a feminist care ethic needs to be central to the ways in which our future health and social care systems are configured. An ‘Ethic of Care’ is difficult to define, but some of the characteristics include attentiveness, responsibility, competence, and responsiveness, and these to be integrated through all the phases of care, from organising and doing care, to receiving feedback (Tronto, 1993). The care ethic, according to this approach, is a practice which is possible, in a society which has a strong sense of justice, open discussion, and one which acknowledges the need to equalise power relationships; therefore, care involves political discussion and consensus (Tronto, 1993). Thus, the value of women’s care work and inequalities of access to resources based on age, gender, ethnicity, and disability must be taken into account in care decision-making (Tronto, 1993).

Since the 1980s, neoliberalism has shaped institutions and justified cuts to public spending, in the NHS and social care in the UK (Simmonds, 2021). Currently, in England—and to some extent the rest of the UK—older adults are treated as care commodities (as beds which are allocated tariffs and then bought and sold in a market) and are dependent on large-scale care systems in which their needs are exploited by multi-national conglomerates (Simmonds, 2021). The care of older people in the UK is not valued or resourced, arguably because women are still providing most of the care for older relatives in the family as well as in outside agencies (Bunting, 2020), and the historical injustices of care work being of low value and feminised have continued. Care systems and nuclear family configurations that rely on the exploitation of women’s labour are ‘unreliable and unjust’ (Care Collective, 2020: 17). This, coupled with endemic ageism in society (Ayalon and Tesch-Römer, 2018), is arguably how neoliberal governments have successfully justified the low value and pay associated with health and care work and the low political priority given to the care of older people.

In conclusion, this perspective paper aims to renew calls to understand one’s relationship to caregiving and receiving as fluid and interdependent, rather than viewing the human condition as a binary between either being dependent or autonomous (Tronto, 1993). Care communities need to be reconfigured to interdependently care for one another (Care Collective, 2020; Simmonds, 2021). These networks do not need to be familiar; they can be intergenerational or community based (Care Collective, 2020). The focus of care provision must be shifted to relational, therapeutic, and reciprocal approaches, which integrate the care ethic characteristics of attentiveness, responsibility, competence, and responsiveness within caring networks (Tronto, 1993; Care Collective, 2020; Simmonds, 2021). There are plenty of examples of alternative care models that have been trialled and have been successful in the UK, yet not widely commissioned, from intergenerational housing schemes to therapeutic care homes and age-friendly cities (see Simmonds, 2021 for further details). I suggest that the pandemic could be a moment in time where the impetus gained from the associated failures of the state is politically harnessed to renew the impetus to design a just and reliable system, which enables ethical care practice.

## Data availability statement

The original contributions presented in the study are included in the article/supplementary material, further inquiries can be directed to the corresponding author.

## Author contributions

BS: Conceptualization, Writing – original draft, Writing – review & editing.
